# Proteomic analysis of human epileptic neocortex predicts vascular and glial changes in epileptic regions

**DOI:** 10.1371/journal.pone.0195639

**Published:** 2018-04-10

**Authors:** Gal Keren-Aviram, Fabien Dachet, Shruti Bagla, Karina Balan, Jeffrey A. Loeb, Edward A. Dratz

**Affiliations:** 1 Department of Chemistry & Biochemistry, Montana State University, Bozeman, Montana, United States of America; 2 The Center for Molecular Medicine and Genetics, Wayne State University School of Medicine, Detroit, Michigan, United States of America; 3 Department of Neurology and Rehabilitation, University of Illinois at Chicago, Chicago, Illinois, United States of America; University of Modena and Reggio Emilia, ITALY

## Abstract

Epilepsy is a common neurological disorder, which is not well understood at the molecular level. Exactly why some brain regions produce epileptic discharges and others do not is not known. Patients who fail to respond to antiseizure medication (refractory epilepsy) can benefit from surgical removal of brain regions to reduce seizure frequency. The tissue removed in these surgeries offers an invaluable resource to uncover the molecular and cellular basis of human epilepsy. Here, we report a proteomic study to determine whether there are common proteomic patterns in human brain regions that produce epileptic discharges. We analyzed human brain samples, as part of the Systems Biology of Epilepsy Project (SBEP). These brain pieces are *in vivo* electrophysiologically characterized human brain samples withdrawn from the neocortex of six patients with refractory epilepsy. This study is unique in that for each of these six patients the comparison of protein expression was made within the same patient: a more epileptic region was compared to a less epileptic brain region. The amount of epileptic activity was defined for each patient as the frequency of their interictal spikes (electric activity between seizures that is a parameter strongly linked to epilepsy). Proteins were resolved from three subcellular fractions, using a 2D differential gel electrophoresis (2D-DIGE), revealing 31 identified protein spots that changed significantly. Interestingly, glial fibrillary acidic protein (GFAP) was found to be consistently down regulated in high spiking brain tissue and showed a strong negative correlation with spike frequency. We also developed a two-step analysis method to select for protein species that changed frequently among the patients and identified these proteins. A total of 397 protein spots of interest (SOI) were clustered by protein expression patterns across all samples. These clusters were used as markers and this analysis predicted proteomic changes due to both histological differences and molecular pathways, revealed by examination of gene ontology clusters. Our experimental design and proteomic data analysis predicts novel glial changes, increased angiogenesis, and changes in cytoskeleton and neuronal projections between high and low interictal spiking regions. Quantitative histological staining of these same tissues for both the vascular and glial changes confirmed these findings, which provide new insights into the structural and functional basis of neocortical epilepsy.

## Introduction

Epilepsy is a neurological disorder that affects about 1% of the population worldwide. It is often called a spectrum disorder because of the large number of different etiologies or ‘brain insults’ that are associated with epilepsy. Similarly, symptomatic seizures can be clinically quite different, depending on where in the brain they arise. While seizures can often be treated symptomatically with anti-epileptic drugs, about 30% of the patients have seizures that are refractory to medications and many of these patients can benefit from a surgical procedure that removes electrically defined brain regions, while largely preserving normal regions [1,2]. The samples removed in these surgical procedures offer an opportunity to use proteomic and other high-throughput approaches on the resected human brain tissues to probe the molecular basis of human epilepsy [3]

The heterogeneity in resected tissues generated from such surgeries has made it hard to use these tissues to study the pathogenesis of epilepsy, in part due to the lack of suitable control samples. Previous studies have used specimens from non-epileptic, age and gender matched individuals as control samples [4,5], but this is far from optimal, since natural biological variation and differences in antiepileptic medication could well mask epilepsy-specific changes. We have developed a Systems Biology of Epilepsy Project to provide a new approach to study human epilepsy. Rather than studying histologically pathological tissues, the Systems Biology of Epilepsy Project focuses on human neocortical tissues that are located by *in vivo* recorded electrical activities, by quantifying the frequency of epileptic potentials between seizures (interictal activity). Omic and histological comparisons are made within each patient, between high- and nearby low-spiking control tissue [6]. This is a powerful experimental design, since high spike frequency in certain brain areas has been found to predict the ictal-onset locations, and resection of the interictal high spike tissue has been associated with good surgical outcomes for seizure frequency reduction [7]. In the present studies, the focus on tissues with abnormal electrophysiology allows for differentiation of proteomic changes specifically associated with epileptic activity and not due to less localized secondary responses, such as inflammation or gliosis.

Using this experimental approach, we have previously found highly consistent changes in gene transcription between regions that produce seizures [8,9] and high levels of interictal spiking [10,11], compared to nearby ‘control’ regions with little to no epileptic activities. Here, we have taken a proteomic approach, using 2-D differential in gel electrophoresis (2D-DIGE), to study six pairs of high and low spiking human neocortical tissues, to determine if there are common proteomic changes as function of abnormal epileptic activity across these patients. The 2D gels have the advantage that they directly display the relative abundances and patterns of protein isoforms that change in the diseased subjects. All of the tissues investigated here were also used to investigate transcriptional changes [10,11]. Our results show consistent changes in proteins that predict decreased glial cells and increased vascularity, as well as a hierarchy of ontological changes that could underlie the development of epilepsy. While some of these changes have been independently found through gene expression studies, many changes implicated here by proteomics are unique, providing independent and added value to our understanding of the epileptic human cortex.

## Material and methods

### Human tissue collection and processing

**Fig 1:**
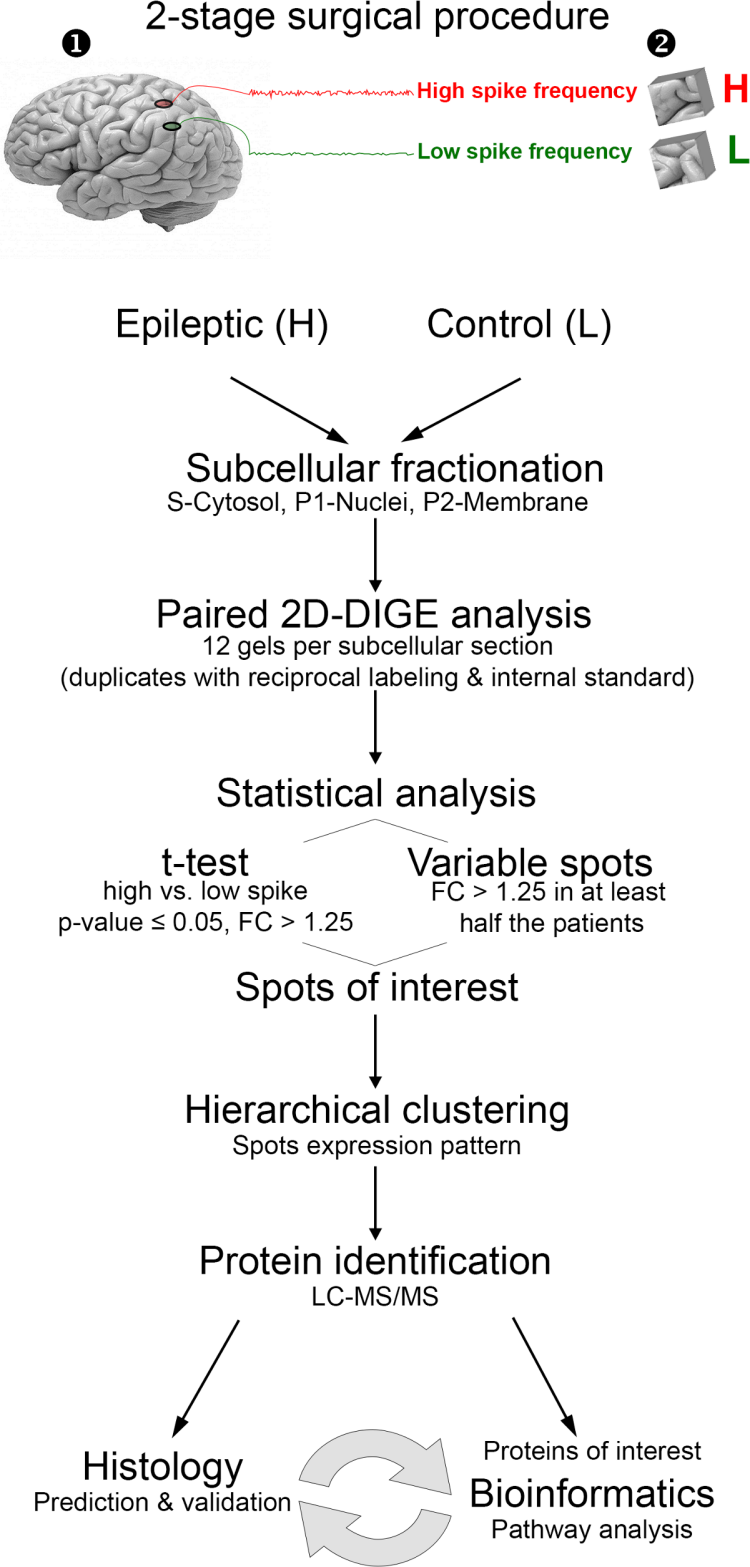
Experiment flowchart. Pairs of tissues from low and high spiking neocortical brain regions are removed as part of the patient’s clinically-determined surgical procedure to treat refractory epilepsy. Each tissue sample is homogenized and separated by centrifugation into three subcellular fractions (Cytosolic, Nucleic and Membrane) and analyzed by 2D-DIGE. Spots found to vary were identified by LC-MS/MS. The proteomic information was used in pathway analyses.

Human neocortex samples were acquired from six patients with refractory epilepsy, written informed consent was obtained from all patients prior to their surgeries through an approved IRB at Wayne State University (Detroit, MI). Only tissues not needed for their clinical care that would otherwise be discarded were used. For minors, signed consent was obtained by parents or guardians. Only tissue regions that were classified *in vivo*, using long-term subdural grid electrical recordings, were used after removal, as part of the patients medical treatment in a two-stage surgical procedure at the Comprehensive Epilepsy Center at Wayne State University, as described earlier [8–11]. Briefly, electrical recordings, taken over several days, were quantified for their intrinsic interictal spike frequency. Regions showing high spike frequency were compared to nearby ‘control’ regions, defined as regions showing no or the least amount of epileptic activity. After excision, the brain samples were further dissected, as described by Loeb [3] and Lipovich et al [10], to provide adequate samples for a range of analyses within the Systems Biology of Epilepsy Project, with plans for future integration of the data (as illustrated in the experimental flowchart in Fig 1). Samples underwent subcellular fractionation to produce three fractions: P1 –nuclear, P2 –membrane, and Supernatant–cytosol, as previously described by Beaumont et al [9]. The Bradford protein assay was used for protein quantification [12]. The fractionated samples were frozen and sent to Montana State University on dry ice for proteomic analysis.

### Protein labeling and 2D-DIGE

Frozen samples were lyophilized at Montana State University and suspended in denaturing buffer (7 M urea, 2 M thiourea, 3% (w/v) CHAPS, 1% (w/v) amidosulfobetaine-14, 10 mM Tris-HCl pH 8.4) to bring the protein concentration to 5 mg/mL. Samples were reduced with 5 mM tributylphosphine for 1 h, followed by alkylation with 10 mM 4-vinylpyridine for 2 h, all at 4°C. The fluorescent dye covalent labeling procedure was done as recommended by GE Healthcare with a few modifications. Briefly, all dyes, Cy2 (GE), Cy3 & Cy5 (synthesized by the Grieco group at MSU) were brought to 400 pMole/μL in dry dimethylformamide, and reacted with the proteins at the ratio 8 pMole dye/μg protein on ice in the dark for 30 min. The reactions were quenched with 1 μL of 100 mM lysine for 10 min. After labeling, reaction tubes were combined to contain 50 μg of Cy3 labeled proteins from a High Spike sample, 50 μg of Cy5 labeled proteins from a Low Spike sample from the same patient and 50 μg of Cy2 labeled, pooled internal standard. The pooled internal standard of each subcellular fraction contained an equal amount of protein extract from High and Low Spike samples from all patients. A technical replicate was prepared from the same unlabeled protein samples, using reciprocal Cy3 and Cy5 dye labeling between High and Low Spike samples. The Cy2, Cy3 and Cy5 labeled samples were combined and brought to 450 μL with re-hydration buffer (7 M urea, 2 M thiourea, 2% (w/v) CHAPS, a trace of bromophenol blue, and 0.5% (v/v) final concentration of 3–11 non-linear ampholines (GE Healthcare)). The solutions were loaded on 24 cm pH 3–11 non-linear IPG strips (GE Healthcare) for passive re-hydration over night at 4°C in the dark. An Ettan IPGphor II was used for iso-electric focusing, with the protocol recommended by the manufacturer. Focused IPGs were agitated for 30 min in equilibration buffer (6 M urea, 375 mM Tris-HCl pH 8.8, 20% (v/v) glycerol, 2% (w/v) SDS), loaded onto 11% SDS gels, and overlaid with agarose. The second dimension SDS-PAGE was carried out using Ettan DALT twelve System, and the protocol recommended by GE Healthcare.

### Image acquisition and analysis

Fluorescent scanning of the gels was carried out using a Typhoon Trio (GE Healthcare). The scan resolution was 100μm/pixel and the manufacturer’s recommended settings were used. The image analysis was carried out using Progenesis SameSpots software (Nonlinear Dynamics, version 3.1). The three subcellular fractions were analyzed as independent data sets, each set containing 12 gels and 36 analytical gel images at each of three fluorescent colors. Standard settings were used for spot detection, background subtraction and ratiometric normalization. The initial statistical analysis utilized the ANOVA test with p-value < 0.05 and fold change (FC) ≥ 1.25 to detect protein spot expression differences between high and low spike samples of each patient. Spots that were found to have expression differences in 3 or more patients were designated “variable spots”. Ratiometrically normalized spot volumes for each spot were exported for further analysis.

In order to quantify relative concentrations of GFAP in the different patient samples, we added the intensities of all GFAP isoforms together. Using ratiometric normalization the changes in different spots cannot be related to each other, thus we carried out a second analysis, using normalization by the total spot volume on each color image. This second analysis allowed us to unite the data from all three sub cellular fractions for all subjects for analysis. The spot intensity values used for each spot were the average of the normalized volumes of all of the technical replicates.

### Histochemical staining of blood vessels

To quantity the blood vessels we used 3,30-diaminobenzidine (DAB, Sigma) directly on the slides and stopped the staining as soon as a dark coloration appeared. This reaction gives a dark coloration of all the cells and allowed visualization of the blood vessels. by microscopy [11]. The length and density of the blood vessels were measured using Metamorph® 6.2 software (Molecular Devices Inc, CA, USA).

### Protein identification by mass spectrometry

Preparative gels were run to resolve the proteins of each of the subcellular fractions for protein spot picking and mass spectral analysis. The preparative 2D gels contained a total of 500 μg protein, including 450 μg unlabeled fractionated epilepsy brain tissue, plus 50 μg of the Cy2 labeled internal standard, which was used for spot alignment with the analytical gels. After fluorescence imaging, preparative gels were stained with silver Coomassie [13]. Spots of interest were manually excised from the gels and In-gel digested with porcine trypsin (Promega) overnight at 37°C, as previously described [14]. Extracted peptides were analyzed, using one of two LC/MS/MS systems, previously described [14,15] with small modifications. One system was an Agilent 1100 HPLC coupled to an XCT-Ultra 6330 Ion Trap and an in-line Agilent microfluidic Chip LC (43 mm 300A C18 separation column with 40nL trapping column). Peptides were eluted with a 5–95% acetonitrile gradient over 22 min. The second system was an Agilent 1100 HPLC coupled with an 6520 Accurate-Mass Quadrupole Time-of-Flight LC/MS (Q-TOF) and an in-line microfluidic Chip LC, as described above (Agilent Technologies). Peptides were eluted with a 5–50% acetonitrile gradient over 16 min. MGF compound list files were generated, using the default algorithms in DataAnalysis 3.3 (Bruker Daltonics) and Qualitative Analysis B.04.00 (Agilent Technologies) software for XCT and Q-TOF, respectively. We used MASCOT in-house version 2.2 (Matrix Science, London, UK) to query the MS/MS spectra, using the following parameters: database: SwissProt 2012 (534242 sequences; 189454791 residues), taxonomy: human (20317 sequences), enzyme: trypsin, allowing up to 1 missed cleavage, fixed modification: pyridylethyl (C), variable modifications: deamidation (NQ) and oxidation (M). Peptide tolerance was ± 1.2 Da and 30 ppm, MS/MS tolerance ± 0.6 Da for the XCT and 30 mmu for the QTOF. Protein identification was considered significant using the default threshold of p < 0.05. For MS results see supplementary material.

### Western blots

A mass of five μg of protein of the high and low spike samples from the nuclear fraction from each patient, were each labeled with Cy3, as previously described, and resolved on mini 10% SDS-PAGE gels, following the standard Bio-Rad procedure. The proteins were then blotted onto Hybond-P membrane (GE Healthcare), using an XCell II apparatus (Invitrogen), following the manufacturer’s recommended procedure. The membrane was blocked with 5% nonfat dry milk in TBST (20 mM Tris pH 7.6, 140 mM NaCl, 0.1% Tween 20) for 2 h, reacted with monoclonal mouse anti-GFAP (NeuroMab N206A/8) at 1:1000 dilution in TBST for 2 h, washed three times with TBST, reacted with Goat anti-mouse IgG Alexa 488 conjugated antibody (Invitrogen) at 1:2000 dilution in TBST for 2 h, and imaged with the Typhoon Trio (GE Healthcare). ImageJ [16] was used for quantification. Six high intensity Cy3-labeled nuclear fraction bands were used as loading controls.

### Statistical and pathway analysis

We used the lme function in the R package nlme [17] to independently test for significant natural log fold-change (high/low) difference from 0 for each protein spot. A p-value < 0.05 for the two-sided t-test and FC ≥ 1.25 was deemed significant. A total of 34 spots passed the t-test cutoffs out of the 4400 measured spots. Thus, we have not corrected for multiple testing, but many of these spots were independently authenticated. GFAP was validated by Western blots, and excluding two spots with ambiguous MS identifications, all other statistically changing proteins had multiple isoforms, and each isoform independently passed the t-test cutoff or very close to it. Thus, the multiple independent identifications of the proteins in different isoforms strengthened the notion that these protein changes were not found by chance. By implementing the mixed model mechanism of lme, we accounted for the variance structure of technical repetitions that were nested within the values from the individual patients, which were imposed by the reciprocal dye-labeling design of the paired experiments.

Spot of interest (SOI) cluster analysis was carried out using Ward's Minimum Variance Method to perform hierarchical clustering in R [18]. Pairwise dissimilarities (distances) between observations were computed with Cluster Package [19], using the "Daisy" function and Gower's Metric [20].

Data were analyzed through the use of IPA (Ingenuity^®^ Systems, www.ingenuity.com, version 42012434, release date 2017-12-07). The IPA core analysis was applied to the list of SOI with fold change values ≥ 1.3 FC cutoff and default parameters. The enrichment within the nervous system was tested using Fisher’s exact test and predicted the involved pathways for each patient. A comparison analysis across the patient population was used to identify pathways for which involvement was frequently predicted (–log(p-value of pathway) ≥ 1.3, in at least 3 patients).

## Results

### The human epileptic neocortex proteome

Tissues from patients who underwent cortical resections for intractable seizures, consenting to be part of our System Biology of Epilepsy Project, were used for this proteomic study. The patients undergo a two stage procedure that begins with several days of subdural electroencephalography recording, followed by excision of the epileptic foci that often contain nearby, electrically quieter “normal” surrounding tissue, as part of their surgical resections. Only tissues that were part of the planned surgical procedure and resected by the neurosurgeon were used. Pairs of 1 cm^3^ blocks of tissue precisely localized to high and low spiking regions from each patient were further subdivided for histology, genomics, and proteomics, as described [3].

Differential proteomics analysis was carried out after subcellular fractionation and differential fluorescent labeling of High and Low Spiking sample pairs from each of 6 patients (Table 1), using the 2D DIGE method to provide improved sample comparison, protein resolution, sensitivity and increased dynamic range [21]. In total, more than 4,400 protein spots were analyzed in independent data sets from the three subcellular fractions. In a t-test (p<0.05, FC > 1.25), 34 spots were found to change between High and Low Spiking samples. 31 of these spots were identified by mass spectroscopy as originating from 18 gene products, some of which existed in multiple protein isoforms. In high spiking regions eight gene products were up regulated (*SNCA*, *STMN1*, *UGP2*, *DSP*, *CA1*, *PRDX2*, *SYN2* and *DPYSL2*) and ten were down regulated (*GFAP*, *HNRNPK*, *CPNE6*, *CRYAB*, *GNAO1*, *PHYHIP*, *HNRPDL*, *ALDH2*, *GAPDH* and *LASP1*) (S1 Table). Given the wide range of spike frequencies and potential variance in absolute protein expression between patients, we also hypothesized that spots that change between high and low spiking regions within individual patients, but have a high variance between patients (“variable spots”) may be biologically significant and might indicate involvement in an active process or molecular pathway that is changing, but would not meet the criteria to pass a simple t-test for all the subjects. Therefore, we also selected these “variable” spots (FC > 1.25, which were up or down regulated in the high spiking tissue, in at least half the patients) in each sub-cellular fraction. A data set of 397 SOI resulted, including statistically changing (t-test) and “variable” spots from all three fractions. 2D maps of SOIs shown in S1 Fig. These SOI were further analyzed, using clustering methods.

**Table 1:** Patient clinical information and tissue samples used for this study.

Patient	Gender	Age (years)	Spike frequency		Patient histopathology
			High	Low	
ep122	F	15	6	0	Normal
ep132	F	10	116	1	Diffuse subcortical gliosis, inflammation
ep150	M	33	371	115	Normal
ep158	M	1	85	0	Subcortical Gliosis
ep159	M	27	27	2	Mild subcortical gliosis
ep165	F	3	212	56	Mild subcortical gliosis

Note that histopathology of each patient was determined on different tissue than those used for proteomic studies. Only the tissues with normal-appearing histology were used. The gliosis described was most commonly found subcortically in the white matter, not in the gray matter that was used for proteomics.

In cases where a protein displayed a spot train of isoforms, in which each spot had a similar up or down regulated expression pattern, only the most intense isoform spots were picked for mass spectrometry-based protein identification, and the other isoforms in the similarly regulated spot train were assumed to be the same protein. Some SOIs were very low in abundance, while some were not ubiquitously expressed, leading to their apparent absence in the preparative gels. In total, more than 90% of the 397 SOIs were identified as arising from 146 gene products (S1 Table). We did not attempt to characterize the numerous isoform modifications present in the proteins, at this stage.

### Predicted histological changes through hierarchical clustering of protein spot expression patterns

**Fig 2:**
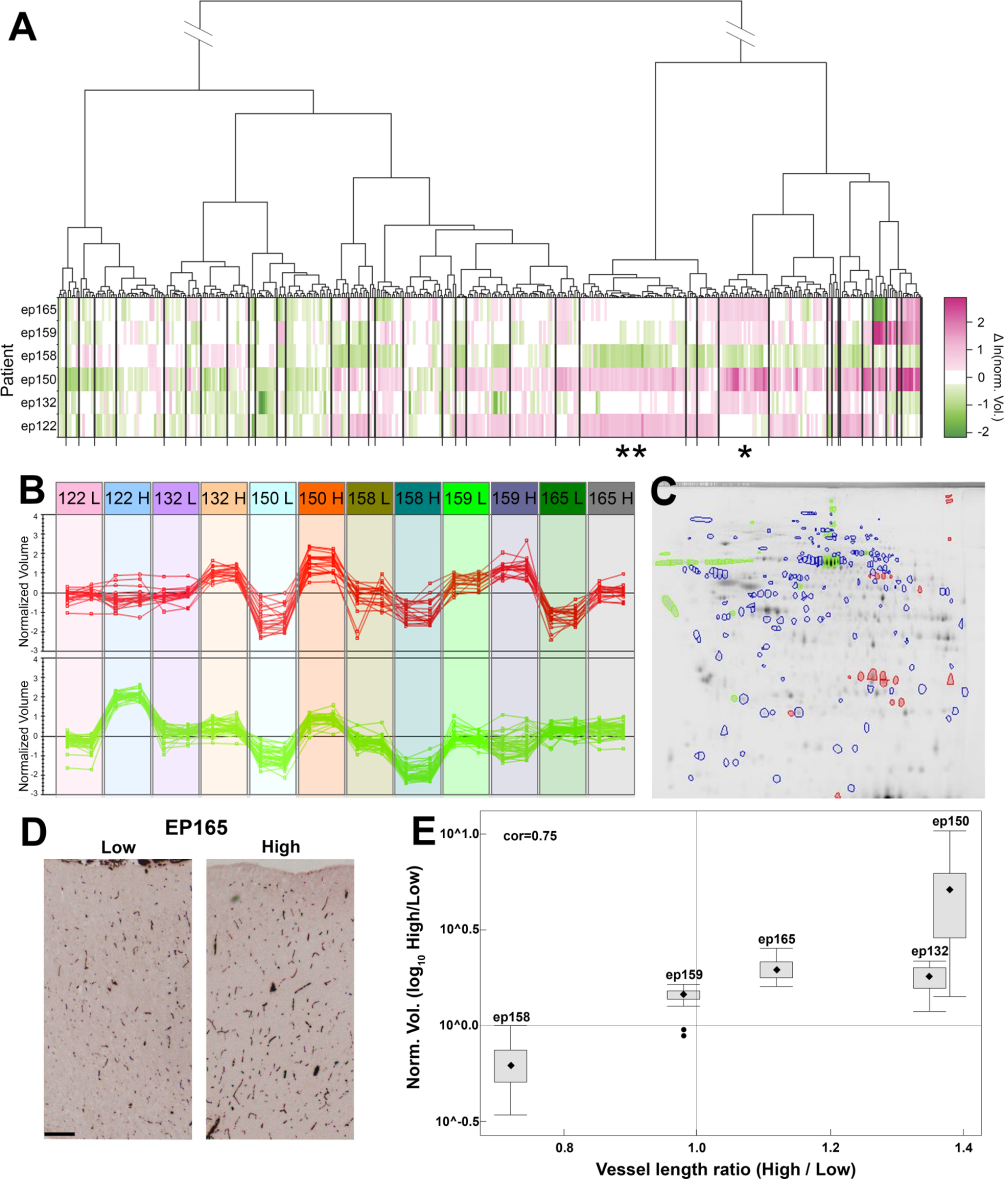
Hierarchical clustering by protein spot expression patterns. (A) Spots of interest (SOIs) from all three subcellular fractions were clustered by expression patterns (level and direction of variation relative to the internal standard) across all samples (dendrogram) and then aligned with the heat map based on their fold changes in SOI (High/Low spiking) by patients. (B) Examples of different expression patterns, which represent the colored spots in the 2D gel image to the right (Fig 2C), grouped together into two distinct clusters. Each of the lines connects a normalized spot volume of technical replicates of high and low spike frequency regions of all 6 patients (the high and low patient sample identifications are in the colored boxes at the top of Fig 2B). (C) 2D gel location of soluble fraction erythrocyte protein spots (red) and extracellular proteins (green) represented in clusters * and ** respectively in Fig 2A. Note: horizontal spot trains are largely due to several isoforms of single proteins. (D) Representative histochemical staining comparing the blood vessel density in high and low spike frequency samples from the same patient demonstrate the predicted increase in vascularity. The scale bar in D indicates 0.5 mm. For detailed method and analysis see [11]. (E) Box and Whisker plots [22] of the changes in vascularity predicted by the ratio (High/Low spiking) of 23 protein spots in the erythrocyte cluster * (in Fig 2A) correlated with blood vessel length ratio (r = 0.75) The ep122 high spiking tissue sample was not available for staining, leading to the exclusion of patient ep122 from this analysis.

In human studies, biological variation is typically conceived of as an inherent limitation which might mask disease or treatment affects. However, in this part of our study, where our focus was to find proteins which change their level of expression in the same direction relative to the internal standard across all the samples, biological variation has been beneficial. The use of a pooled internal standard control, containing proteins from all samples, facilitated the comparison of spot expression levels across the whole dataset. To provide more expression data points and higher pattern specificity (similarities in relative expression), samples were treated independently, regardless of patient and spike frequency, and variation in expression between and within patients were weighted equally. The added variation revealed unique protein expression patterns and enabled better separation of co-expressing protein groups (Fig 2). The SOIs were clustered and visualized on a dendrogram, according to how the level of their expression varied across the 12 samples (6 patients x 2 spike frequencies). SOIs that showed similar levels of variation for all samples were clustered together (Fig 2A and 2B). Some spots clustered more tightly together, sharing short vertical branches on the dendrogram. These tight clustering observations were also validated by Leave One Out Cross Validation.

It is important to emphasize that the similarities in expression pattern of a group of spots, across patients, indicates that these changes should be treated as a single finding, allowing for reduction in complexity of the dataset. For example, groups of similarly expressed isoforms, of a single protein, which are changing in expression across the samples, will have essentially the same expression patterns (Fig 2B and 2C). Another example is a protein complex that is expressed at different levels in different samples, since the subunits of the protein complex will have the same expression patterns (change in the same direction). It is likely that the tightness of the cluster will decrease with increasing numbers of gene products in the complex, due to multiple possible regulations and involvement of proteins which are ubiquitously expressed. However, due to the high biological variation between individuals, and the resulting enhanced clustering power of this approach, we were able to apply the same logic to identify clusters that we hypothesize represent changes in the phenotype of a given cell type, within the neocortical tissue. Particular cell types were predicted, based on known cell-specific protein spots within a given group, that were identified using mass spectrometry. Detailed information on protein identification by fraction and spot is given in S3 Table, in the SOIs cluster assignment in S2 Table, and on MS identification of SOIs by Mascot, as provided in S4 Table.

Several histological changes were predicted from the groups of proteins found by hierarchical clustering. Following the right most branch of the four main dendrogram branches in Fig 2A (see S2 Fig for enlarged dendrogram and cluster number designations), we found several indicators of blood, including platelets—indicated by a fibrinogen cluster (cluster 37), that includes multiple isoforms of two of the fibrinogen subunits (FGB, FGG). Evidence for fibroblasts, smooth muscle or possibly pericytes, was indicated in this branch by co-expression of TPM1, TPM2 and TAGLN (cluster 34). The presence of plasma in clusters 28 & 33 was indicated by albumin and components of high-density lipoprotein (APOA1, APOD). Finally, the presence of erythrocytes was indicated in cluster 27 primarily by hemoglobin, but also by a cluster of proteins, all of which are present in high abundance in red blood cells (HBA, HBB, catalase, CA1, BLVRB) [23–25]. Since all tissue sample surfaces were rinsed to remove blood before homogenization, we attributed changes in blood levels to changes in vascularity in the tissues. The relative change in vascularity in the brain tissue, predicted by the erythrocyte cluster, was validated by histochemical staining (Fig 2D and 2E).

The next dendrogram branch, second from the right in Fig 2A (cluster number 24 in S2 Fig), contains mostly albumin isoforms, as well as other extracellular proteins. The distinct protein expression patterns between the extracellular and erythrocyte proteins (Fig 2B and cluster numbers 24 and 27 in S2 Fig) indicate that the changes in albumin levels are not solely due to changes in vascularity (circulating serum albumin), and suggested the presence of additional albumin in the extracellular space.

**Fig 3:**
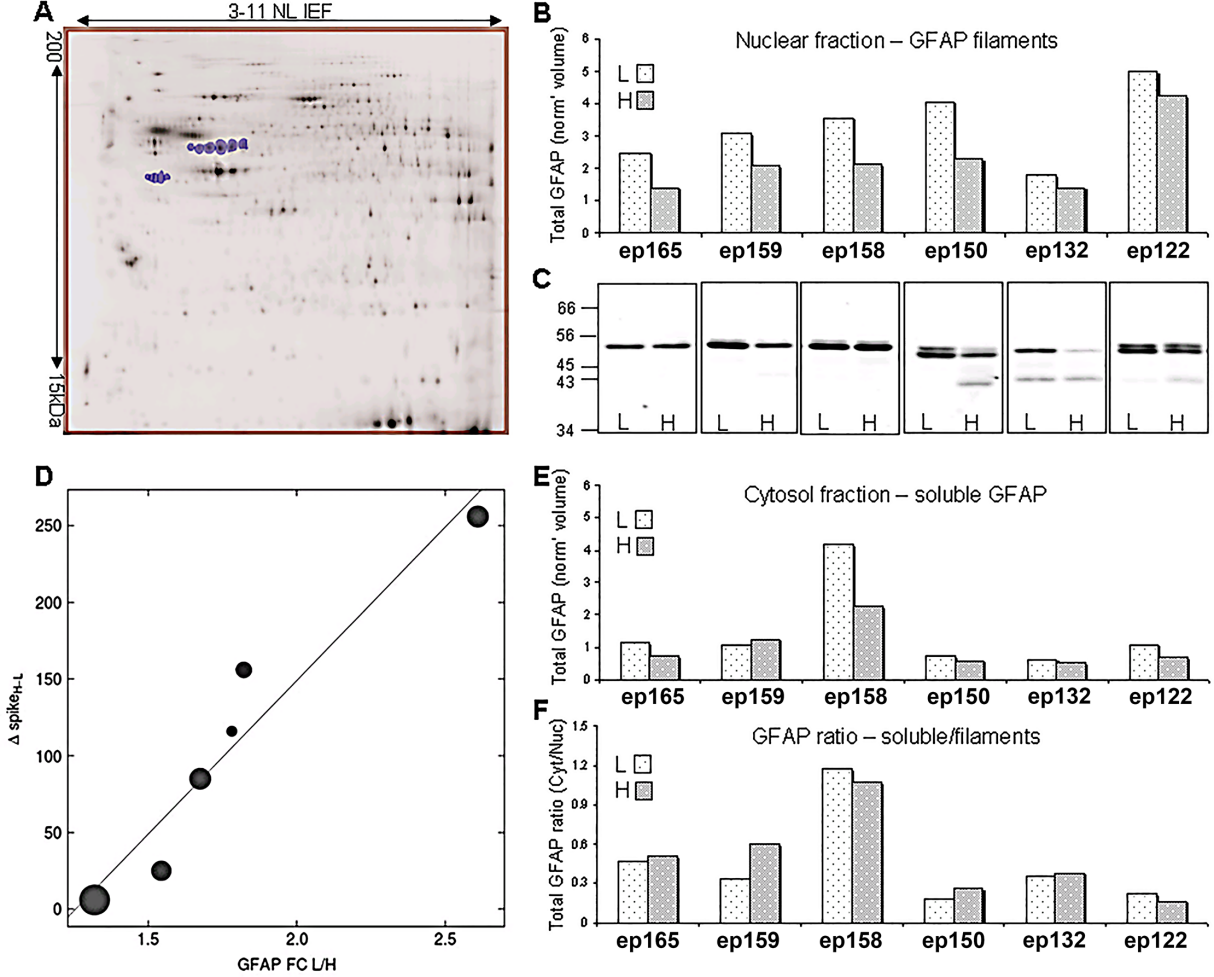
GFAP quantification studies. (A) 11 GFAP SOIs are the blue marked spots in the 2D SDS-PAGE image of the P1 fraction, which were used for quantification of total GFAP. (B) Total GFAP as the sum of total image intensity normalized volume (non-ratiometric) of nuclear GFAP spots shown by patient and spike frequency (L-low, H-High). GFAP is down regulated in high spiking samples (paired t-test for all 11 spots or upper higher MW (~50kDa) 6 spots, p-value 0.002, average FC 1.53 or 1.79, respectively). (C) Western blot analysis for all nuclear fraction samples with a monoclonal anti-GFAPα antibody, validated the down regulation of GFAP in high spiking samples (p-value 0.047). (D) Correlation of fold change of nuclear fraction 50kDa GFAP (upper 6 spots) (L/H spike ratios, circles are sized by the weighted total intensity) with differences in spike frequency for individual patients, Δspike = H-L.Y = 200X – 251, r = 0.96, p-value 0.002. (E) Sum normalized volume of cytosolic GFAP spots by patient and spike frequency. (F) Cytosol to nuclear total GFAP ratio (11 spots). Note—for GFAP quantification, fluorescence spot intensities were normalized to total fluorescent protein signals in each gel image. Values are given as the sum of relevant spot normalized volumes in millions of volume units.

Many of the identified SOIs were related to cytoskeleton proteins, suggesting structural or migrational changes in affected cells. Dominating this group of proteins, with statistically significant down regulation in the high spiking regions in all fractions for all patients was GFAP (~50kDa): a commonly used astrocytic marker. As one of the major astrocytic intermediate filaments, the cytoskeletal GFAP mostly pellets in the nuclear fraction. The total nuclear fraction GFAP SOIs (the sum of 6 spots of ~50 kDa + 5 spots of 38–40 kDa) normalized spot volumes (Fig 3A), confirmed the decrease of GFAP in the high spiking regions (paired t-test p-value 0.002, average FC 1.53, Fig 3B). Western blot analysis validated the decrease of total nuclear GFAP in high spiking tissues (p-value 0.047, Fig 3C). The spot group of lower molecular weight (LMW, 38–40 kDa) GFAP presented a different trend of expression across the patients. These LMW GFAP spots were mostly up-regulated in high spiking regions and notably clustered with blood proteins, as shown in cluster 32–33. The six 50 kDa GFAP isoforms in the P1 (nuclear fraction) added together demonstrated a stronger down regulation in the high spiking regions (p-value 0.002, FC 1.79), if the LMW GFAPs are excluded from the analysis. Interestingly, the increase in total GFAP fold change (L/H spike) of the 50kDa isoforms was strongly correlated with increased spike frequency (frequency difference High-Low = Δspike), as shown in Fig 3D. The linear correlation coefficient was r = 0.96 with a p-value 0.002, using the Pearson product-moment correlation coefficient. Alternatively, we evaluated the non-parametric association and found a Kendall tau coefficient of r = 1, p-value = 0.003 for the data plotted in Fig 3D.

The total GFAP changed between high and low spike regions only in the nuclear pellet fraction and did not significantly change in the P2 and Cytosol fractions. The total GFAP in the Cytosol fraction (Fig 3E), although still mostly higher in low spiking regions, demonstrated a very different expression pattern between the patients, when compared with the nuclear fraction (Fig 3B & 3E). The levels of cytosolic GFAP were more in agreement with the level of gliosis, which was assigned to the patients by the pathological analysis (Table 1), but was not a good predictor for astrogliosis status since much of the gliosis seen was subcortical in white matter, not in the gray matter where our proteomic analysis was performed. The GFAP soluble pool (in the cytosolic fraction) is presumably related to the level of GFAP filaments (nuclear fraction) and might be affected by pathological conditions [26]. Therefore, we evaluated the ratio of total cytosolic to total nuclear GFAP concentration and found the ratio to be a good predictor for the patient’s astrogliosis level, as can be seen by comparing the patient histopathology in Table 1 to the data in Fig 3F. This analysis found that tissues with no apparent histopathology had a soluble GFAP concentration between one-quarter to one-fifth of the concentration in the nuclear fraction. Diffuse and mild levels of gliosis were associated with increased shift to soluble GFAP, until the soluble and insoluble GFAP concentrations were about equal for the patient with the most pronounced gliosis.

### Bioinformatic analysis of proteins of interest

The 397 SOIs originate from 146 gene products. Multiple spots for the same gene product might arise from alternative splicing and/or posttranslational modification, as well as a SOI occurring in more than one subcellular fraction. In this exploratory phase of the project, we have identified candidate proteins for further study and did not investigate specific modifications of gene products.

Description of the 146 proteins of interest is given in S1 Table and their FCs are shown with color codes by patient. Since multiple isoforms were often summed to a single gene product, the protein fold change represents the average expression of sets of isoforms for each patient. Therefore, the absence of a significant, net fold change in at least three patients of a given protein with multiple SOIs is likely to indicate approximate cancelation of FC by differential post-translational modifications. An exception was made with regard to analysis of GFAP, which had two very distinct groups of multiple isoforms. First, the spot train having the expected MW (~50kDa), including the expected pI for full length GFAP. Second, several isoforms, identified as GFAP by MS, are modified with lower than the predicted MW and different levels of acidic shift in isoelectric point. These were analyzed separately as two GFAP products, GFAP ~50kDa and GFAP LMW (low molecular weight).

**Fig 4:**
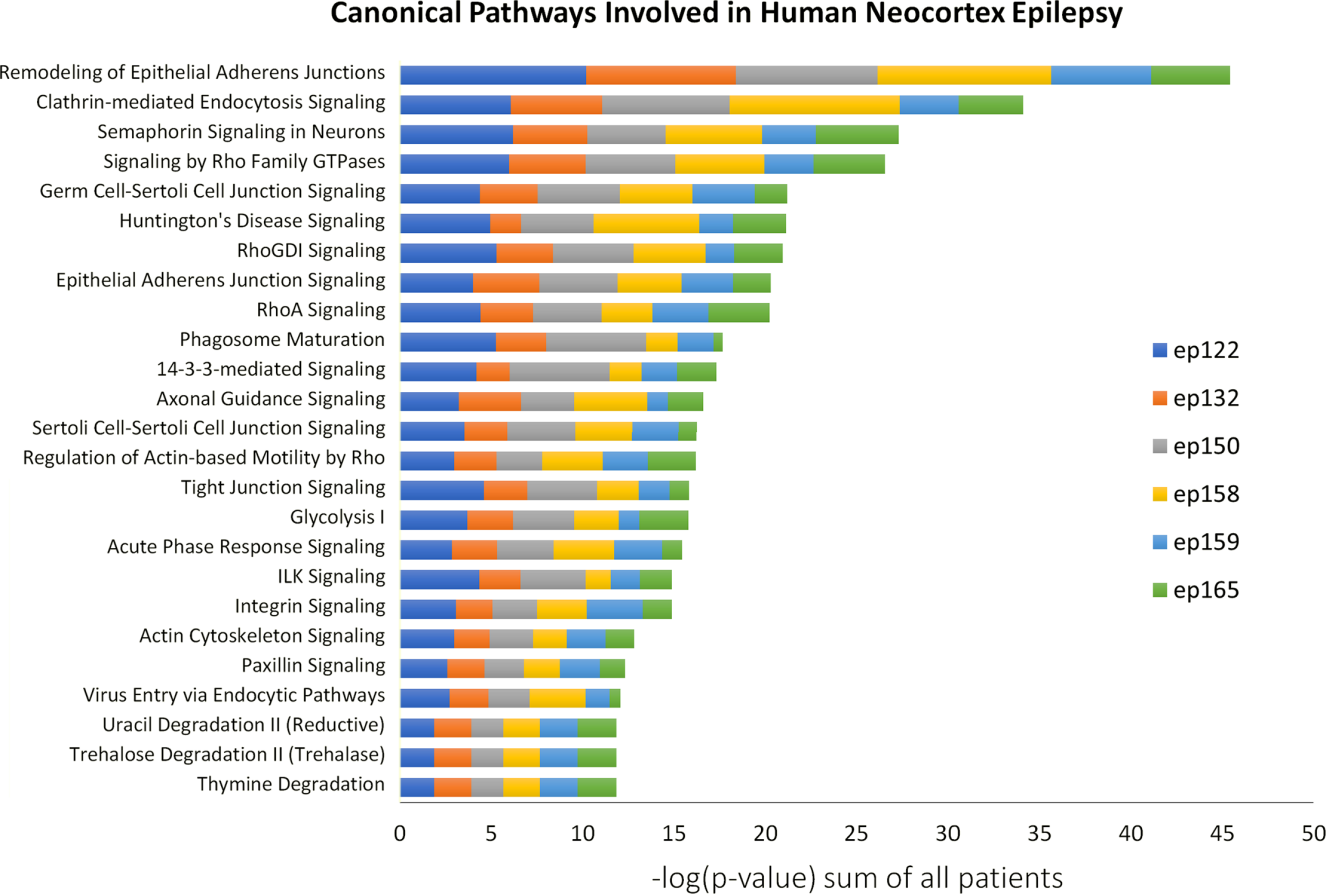
Top 25 canonical pathways involved in epileptic brain regions are predicted by Ingenuity Pathway Analysis ranked by significance. The sum of the logarithm of the p-value for all the patients and for each canonical pathway found is ranked from the most significant to the less significant. The contribution that each patient has on the sum is represented by color codes.

Ingenuity Pathway Analysis (IPA) was used to predict involvement of Canonical Pathways for each patient. The differential expression and biological involvement of the erythrocyte proteins in relation to changes in vascularity, was already addressed. Therefore, erythrocyte proteins were removed from the analyzed genes (unless the proteins were also expressed outside the erythrocyte cluster). After considering the differences in expression patterns of the blood components, as well as their size and likelihood of leaking out of the vessels under pathological conditions, the rest of the blood components were included in the analysis, since they might have interactions or cellular effects exterior to the blood vessels. Common involved pathways across the patient population were found using IPA comparison analysis. Pathways predicted to be involved in at least three patients were considered. The top 25 ranking canonical pathways involved in human neocortical epilepsy, and the relative contribution of each patient to the score, ranked by p value, are shown in Fig 4.

## Discussion

Surgical resection of electrophysiologically characterized brain tissue from patients with refractory partial epilepsy can be highly beneficial for reduction or elimination of seizures [27,28], and provides a unique opportunity to study the molecular and histological changes associated with abnormal electrophysiological properties of epileptic human neocortical brain regions. The Systems Biology of Epilepsy Project is taking advantage of this opportunity to generate a clinical and multi-omics database to further our knowledge of the epileptogenic process and advance the search for better treatments [3,6]. In the present study we used differential fluorescent labeling in 2D-DIGE to resolve and compare protein expression and protein modifications between high and low spike frequency tissue sample pairs from each patient. In the initial differential analysis we identified a set of proteins of interest, suggesting that these proteins might be involved in the cellular changes that lead to epilepsy. Subsequent analysis focused on attempts to relate this set of proteins to epilepsy-related biological changes. We took advantage of the natural biological variation between patients as well as natural, or epilepsy induced variation within the samples of each patient, using hierarchical clustering, to allow the separation of groups of co-expressing protein spots that predicted histological changes.

### High interictal spike frequency is associated with vascular modification

Many of the changing proteins, when clustered by their expression patterns, suggest significant increases in blood vessels in high spiking neocortex. Although all but one (CA1) of the individual protein changes did not quite reach statistical significance alone, the group of proteins in the blood cluster suggested increased vascularity in high spiking samples in four of the six patients. Patient ep122 showed no change and patient ep158 showed an opposing trend, for this cluster, as well as in most of the changes in the dataset. Since patient ep158 was only one year old, his brain is in a very different developmental stage than the other patients, which might be expected to affect many of its “normal” and “epileptic” features.

The process of angiogenesis is associated with brain injury [29,30] and has previously been described in temporal lobe epilepsy [31], with an association to increased blood brain barrier (BBB) permeability [32,33]. Here, for the first time using precisely mapped cortical regions with more and less epileptic activity, we predicted increased vascularity by both proteomic and previous transcriptomic and metabolomic studies [11,34] and these findings were validated using histochemical staining [11]. Together, this work suggests that angiogenesis is closely associated with interictal high spiking neocortical tissues and may play a role in metabolic changes linked to increased cortical activity. Given the correlative nature of these human studies, we cannot say whether the vascular changes cause the epileptic activity or result from increased activity. The consistent finding with all of our transcriptomic, proteomic and metaobolomic studies, however, suggests a clear correlation between epileptic spiking and increased vascularity.

These findings also demonstrate the power of protein expression clustering to interpret proteomic studies. Although carbonic anhydrase 1 (CA1) was statistically up-regulated in high spiking regions, its correlation with hemoglobin levels and other proteins highly abundant in erythrocytes, led us to interpret CA1 up-regulation and the erythrocyte protein cluster as part of increased vascularity. You et al [35] suggested four proteins (hemoglobin, catalase, peroxiredoxin and carbonic anhydrase I) as markers for blood contamination in proteomic analysis of cerebrospinal fluid. It is important to note that, because we use a paired analysis from high and low spiking tissues that were resected in the same manner, the increase in erythrocyte proteins were not due to blood contamination of the tissues, but rather to the observed increased in vascularity in the high spiking regions. We also suggest that the similar expression pattern of most of these proteins, with the addition of biliverdin reductase B (BLVRB), defines them as erythrocytes markers. It is important in gel-based proteomic studies, to use several blood marker proteins since hemoglobin isoforms run close to the electrophoretic front and may be excluded from the analysis. In a gel-free proteomic study, Visanji et al [36] also found increased expression of catalase and CA1 in human epileptic brain, compared with controls, and localized both proteins to astrocytes. In addition to CA1 and catalase, they found seven hemoglobin isoforms, albumin, and alpha-2-macroglobulin to be up-regulated in epileptic brain [36]. They suggested catalase and CA1 had epileptogenic roles in response to oxidative stress (catalase) and support to carbonic anhydrase 2 pro-apoptotic/epileptogenic role (CA1). Although these mechanisms cannot be ruled out, our proposed erythrocyte protein cluster marker and correlation with histological measurements of increased vascularity provides an alternative explanation. We suggest that the protein changes found by Visanji et al [36] are fully consistent with increased vascularity in the epileptic brain.

The astrocytic foot is a structural component of brain vasculature, which can explain the localization of catalase and CA1 to astrocytes. The hemoglobin isoforms, catalase and CA1 are the most abundant proteins in erythrocytes [25]. Furthermore, CA1 expression is limited to erythrocytes and the gastrointestinal tract [37]. Additionally, alpha-2-macroglobulin and albumin are two major carriers of proteins in the serum [38,39]. Taken together, our alternative explanation of increased vascularity in the epileptic tissues, accounts for changes in levels of eleven of the twelve proteins found to change by Visanji et al [36].

### High interictal spike frequency is associated with decreased GFAPα

Reactive astrocytes over-expressing GFAP (gliosis) is a hallmark of posttraumatic brain injury and can be see in hippocampal sclerosis, associated with epilepsy [40]. GFAP is the main intermediate filament in astrocytes, its expression increases as a function of age and is affected by multiple pathological conditions [41]. GFAP is normally found primarily in the water-insoluble subcellular pellet fraction [42], as a polymer, with a small amount in the soluble protein fraction. However, astrogliosis increases the proportion of soluble GFAP [26]. We evaluated the relative GFAP concentration in both the cytosolic and nuclear fractions and found that the ratio of soluble to insoluble GFAP correlated with levels of gliosis from the pathological assessment of the patients (Table 1), regardless of spike frequency.

Surprisingly, the 50kDa GFAP, as well as total nuclear GFAP, was down regulated in high spiking tissue of all the patients, compared with low spiking tissue from the same patient. Furthermore, the fold change decrease in GFAP was strongly related to the level of spiking, suggesting a causal relationship between the two phenomena. In addition to the unexpected decrease in GFAP, α-B-crystallin (CRYAB), another protein whose over-expression is a marker of epileptic foci [43], was found in our study to be down regulated in the P2 subcellular fraction of high spiking tissue. CRYAB is small heat shock protein, known to be strongly induced in reactive astrocytes [44]. CRYAB has a role in the anti-inflammatory response, and an anti-apoptotic role in astrocytes [45]. CRYAB is also known to regulate GFAP assembly and to co-aggregate with it in astrocytic inclusions [46]. It is unclear whether the changes in expression of the two proteins are related. It is possible that the co-reduction is due to a CRYAB & GFAP complex, to a reduction in reactive astrocytes, or to astrocytic apoptosis that may be mediated by the lack of protective CRYAB. The decrease in these two epilepsy foci markers in the high spike tissue, suggests that the cellular changes associated with abnormal electrophysiology are separate from the less localized cellular changes, such as astrogliosis, which are likely to be associated with general trauma or stress. One possible explanation is that more severely damaged cortex with more gliosis is less able to generate epileptic discharges than nearby areas without extensive gliosis and more normal neuronal connectivity.

Several GFAP splice variants have been described in the literature [41], some of which have been found to be differentially expressed in epileptic lesions [47,48]. However, our MS analysis of 2D gel electrophoresis spots, in agreement with 1D western analysis using a specific GFAPα monoclonal antibody, indicates that the canonical 50kDa GFAPα is the variant in the SOIs examined here. While the 50kDa GFAPα was down regulated in high spiking tissues, the insoluble low molecular weight (38–40 kDa), modified GFAPα products had a mixed expression pattern and clustered with the blood proteins. Although the exact nature of this GFAP cleavage process was not studied here, the co-expression with the blood proteins might indicate that insoluble 38–40 kDa GFAPα is an indicator for perivascular astrocytes, or specific GFAP modifications that are associated with the astrocytic foot process, which is an integral component of the blood brain barrier [49].

An increasing body of evidence implicating astrocytic dysfunction in epilepsy has been described [50–52]. Witcher & Ellis [53] addressed a paradox in temporal lobe epilepsy, of increased GFAP and decreased glutamate transporter EAAT2, which is predominantly expressed in astroglial cells and throughout the astrocytic membrane. They consolidated these co-occurring changes with the morphological interpretation of “…decrease in non-GFAP containing perisynaptic astrocytic processes…” Indeed, GFAP might not be an ideal marker for non-reactive astrocytes, since it is not expressed throughout the astrocyte cytoplasm and is not detectable in fine astrocytic processes [54]. In epilepsy models, expression studies of key astrocytic proteins such as aquaporin-4 and inwardly rectifying potassium channel Kir4.1, that are suspected to be epileptogenic, revealed reduction in their immunoreactivity, specifically in cortical (and not hippocampus) astrocytic processes [55,56]. Rakhade & Loeb [57] found that the glutamate transporter EAAT2 was down-regulated in human neocortical epileptic foci in experimental settings comparing high and low spiking regions, similar to our study. Thus, the decrease in GFAP in high spiking regions in our study supports a hypothesis that GFAP positive astrocytes are decreased. In addition, ALDH1L1, an alternative candidate for an astrocytic marker [54], also exhibits a decrease in our dataset (p-value = 0.013, FC = 1.18). One of the challenges of this type of study is that it is difficult to deduce whether a particular change in epileptic brain regions contributes towards hyperexcitability or may be compensatory or protective. Since all of the patients in this study failed medical management (were pharmacoresistant), one can argue that if compensatory, the changes were still not sufficient to prevent seizures. Additional studies are needed to more fully characterize the nature of the changes in GFAP positive astrocytes, whether they are due to a reduction in the number of cells or to a change in their morphology and whether differences in their expression are potentially protective against epileptic activities.

### Canonical pathways involved in refractory neocortical epilepsy point to changes in the blood brain barrier and synaptic plasticity

Knowledge-based software, which predicts the involved pathways from lists of changing proteins, is a powerful tool in the complex field of neuroproteomics [58]. We queried Ingenuity Knowledge-Base Ingenuity Pathway Analysis (IPA) to predict the pathways involved for each of the patients and to identify the common, probable pathways that are associated with biological changes in the high spike frequency tissues.

Many of the identified common pathways partially overlap at the molecular level and share some of the same protein networks. For example, we have identified changes in several protein components of the adherens junction (AJ), which pointed to several of the proposed signaling pathways. IPA identified “Remodeling of epithelial adherens junctions” and “Epithelial adherens junction signaling”, as shown in Fig 4, that can also indicate changes in AJs of endothelial cells, and in our study appears to indicate changes involved in blood vessels or BBB. This notion is supported by other signaling pathways identified by Ingenuity analysis in our study, “germ cell–sertoli cell junctions signaling” and “sertoli cell–sertoli cell junction signaling”. Sertoli cells are obviously not part of this neurological system, but the blood testis barrier and the BBB share some structural and protein component similarities. For example, components of tight junctions, AJs and gap junctions, which together generate the non-fenestrated barrier between compartments [59,60]. AJ changes in the BBB can affect its permeability [61] and are also related to endothelial cells motility and angiogenesis [62,63], re-enforcing our focus on increased vascularity and BBB dysfunction, as was also suggested by the hierarchical clustering of protein expression patterns. An alternative interpretation to AJ changes in the nervous system can be due to re-modeling of synaptic junctions, where the synapses are held together by AJ’s [64], and could change as part of epilepsy related plasticity changes.

“Acute phase response signaling” was another pathway suggested by our study. Although this pathway might have relevance to epilepsy, our study, due to its experimental design, lacks the potential of finding systemic changes (in the blood), such as the acute phase response. However, this pathway, which is represented by a group of modulated plasma proteins, might be mistakenly indicated in cases where there is increased vascularity or BBB dysfunction. Several studies address dysfunctional BBB in epilepsy [65–69]. Increase in extracellular space albumin is assumed to be the result of a leaky BBB [69,70]. Therefore, extracellular proteins changes (clusters 24–26) might be interpreted as further evidence to a leaky BBB in human epileptic neocortex.

“Semaphorin signaling in neurons” and “axonal guidance signaling” found in our Ingenuity analysis, have a high degree of component overlap and suggest fundamental changes in synaptic plasticity in human epileptic neocortex. In a meta-analysis of refractory epilepsy transcription, Mirza et al [71] also found these pathways to be up-regulated. We have identified several proteins involved in these pathways, including multiple isoforms of the collapsin response mediator protein (CRMP) family. The CRMP family is homologous to dihydropyrimidinase, an enzyme responsible for uracil and thymine degradation, consistent with the identification of these degradation pathways in our analysis. Of potentially great interest is CRMP2 (formally, dihydropyrimidinase-like protein-2 or DPYSL2), which we found to be up-regulated in high spiking tissues. CRMP2 is the most abundant and studied member of the CRMPs. CRMP2 is known to have multiple neurological roles, and to be involved in several neuropathological conditions [72]. The CRMP2 antagonist, Lacosamide, is specifically used to treat refractory partial epilepsy seizures, by mechanisms that might involve stabilization of neuronal firing through regulation of voltage-gated sodium channels, or inhibition of abnormal axon sprouting [73–75].

### Biological significance of statistically changing proteins

Understanding the biological significance of the change in a single protein in a proteomics study can be challenging. Therefore, we combined the statistically changing protein spots and the commonly changing spots ('variable spots') for hierarchical clustering and pathway analyses. In addition to these histological modifications and cellular pathways involved in high spiking tissues there were a number of statistically significant individual proteins that warrant further discussion. These include the guanine nucleotide-binding protein G(o) subunit alpha (GNAO1), and LIM and SH3 protein 1 (LASP1). Both proteins are involved in multiple cellular pathways. Our study did not elucidate a single pathway which clearly indicates the biological significance of the down regulation of GNAO1 and LASP1 observed in high spiking tissue. However, we demonstrated that the expression patterns of GNAO1 in the P2 fraction and LASP1 in the P1 fraction have parallel patterns of expression as the insoluble 50 kDa GFAP and CRYAB isoforms (see clusters 1 & 13 respectively in S1 Table). Since GFAP and CRYAB changes were attributed to changes in GFAP positive astrocytes, the changes in GNAO1 and LASP1 expressions may also be of astrocytic origin.

Two neuronal phosphoproteins, alpha synuclein (SNCA) and synapsin II (SYN2), were up-regulated in high spiking tissues. Both proteins might be involved in multiple pathways and processes. Neuritogenesis and synaptic transmission are of special interest, since both of these proteins localize to the presynaptic terminal and interact with cytoskeletal proteins in the nerve growth cone [76,77]. SYN2 has been implicated previously in epilepsy, since certain genetic variants of this protein contribute to the predisposition to epilepsy and its differential effect on inhibitory and excitatory synapses [76]. Another phosphoprotein stathmin1 (STMN1), which is involved in microtubule regulation, was found here to be up-regulated in high spiking tissues. Zhao et al [78] found over-expression of STMN1 in the neocortex of patients with intractable temporal lobe epilepsy, when compared with neocortex of normal temporal lobes. They used immunoassays to localize the changes in STMN1 expression to neurons and suggested that STMN1 participates in neurite sprouting and synapse remodeling. Taken together, our results suggest a heightened level of neurite sprouting and synapse remodeling in the high spiking brain areas that could be linked to the development of hypersynchrony. Our recent studies support this interpretation, showing quantitative, superficial layer-specific increases in presynaptic nerve terminals in epileptic brain regions [9], however, at the same time in another study, we discovered focal reductions of synaptic complexity in deeper layers of high spiking regions called microlesions [11].

Remarkably, transcriptional profiling of the exact same brain regions that we used for the proteomic analysis described here led us to overlapping as well as completely different pathways [9–11]. In both analyses, an increase in vasculature was in common, however the most salient transcriptional pathways centered around MAPK/CREB signaling, which did not come up in the proteomic analysis. One likely explanation is that our proteomic approach using 2D gels works best for higher abundance structural proteins, such as those in astrocytes and blood vessels, rather than lower abundance signaling and transcription factors that may be detected in transcriptional profiles. Given the complexity of the 6-layered neocortex and possible alterations in epilepsy, further studies will be needed to understand how and where posttranscriptional and posttranslational modifications are linked to changes in the cytoarchitecture, that produces epileptic brain regions.

## Conclusions

This study is the first to correlate proteomic changes directly with electrical brain activities recorded *in vivo*, allowing for the unbiased identification of protein changes specifically associated with human epileptic activity. We combined proteomic, statistical and bioinformatic tools to predict and then validate histological changes and cellular pathways associated with the abnormal electrophysiology. We found a decrease in astrocyte markers and an increase in vascularity in the high spiking regions to be a common histological change, that we also previously found in both transcriptomic and metabolomic studies [11,34]. Furthermore, the increase in spike frequency in different patients was strongly related to the level of decreased astrocyte marker GFAP, suggesting causality between astrocytic reduction and abnormal electrophysiology.

Contrary to what is thought for forms of hippocampal epilepsy, where there is a diffuse increase in astrogliosis, our study suggests brain regions with increased astrocytes actually have less epileptic firing. The strong correlation of the reduction of reactive astrocytosis (marked by GFAP) in higher spiking regions, suggests that these reactive astrocytes may protect the neocortex from epileptic discharges, rather than cause them. While this type of analysis has not yet been done for the hippocampus, our findings suggest that local regions of the hippocampus with extensive gliosis may not in fact be more epileptically active as nearby hippocampal areas with less gliosis.

The ongoing integration of the proteomic data with the clinical, histological and other high-throughput studies of Systems Biology of Epilepsy Project is leading to the discovery of cellular and molecular interactomes of human epileptic neocortex. Such an interactome can deepen our understanding and derive new, knowledge based, hypotheses for epileptogenic mechanisms and therefore possibly lead to new therapeutic opportunities.

## Supporting information

S1 Fig 2D maps of all spots of interest (SOIs). Each image represents 2D-PAGE of proteins from subcellular fractions using pH range of 3–11 (non-linear) and MW range 200-15kDa. SOIs are marked by blue perimeter and spot number. Note—the SOIs numbers in the cytosolic fraction are split across two images (CytA & CytB) in order to allow their visualization. P1 is the nuclear fraction and P2 is the membrane fraction. CytA and CytB are identical images of the cytosolic fraction used to accommodate different SOI numbers.
(TIF)

S2 Fig Spots of interest (SOIs) dendrogram. Visualization of hierarchical clustering of 397 SOIs by their expression patterns. The dendrogram was divided into two main branches (A & B) and an enlarged image of each branch is shown. An arbitrary distance cutoff was used to separate the dataset to 37 groups of spots.
(TIF)

S1 Table 146 Proteins of interest, representing 397 protein isoform SOIs. Notes: Number indicates the number of isoforms (spots) of the identified protein, using bin-averages for fold change values. (‡)—indicate a spot with an ambiguous MS identification (see supplementary material for details and definitions). (†)–indicates a spot where the observed MW does not agree with expected MW, implying a gene product modification. ↑ and ↓ indicate a spot with statistically significant up and down regulation, respectively. t-test p-value <0.05 (a) and < 0.005 (b). Patient FC color: dark green ≤ (-1.5), light green ≤ (-1.25), (-1.25) < white < 1.25, orange ≥ 1.25, red ≥ 1.5.
(XLSX)

S2 Table Spots of interest by cluster. SOIs ordered by their dendrogram position. Include the protein assignment and the spot expression ratios (H/L) for each patient. Four levels of identification are used and each one coded by a label: i) '$': identification by MS of a single protein with multiple peptides or the spot has been identified by Western blot. ii) '*': identification of the spot inferred from MS and/or 2D-PAGE information indicate only one potential protein. iii) '/': identification of the spot inferred from MS and/or 2D-PAGE information indicate more than one potential protein. iv) the protein in the spot cannot be identified or predicted by our techniques.
(XLSX)

S3 Table Spots of interest by fraction and spot number. Spots are ordered by fractions and spot number, including protein assignment and description. Four levels of identification are used and each one coded by a label: i) '$': identification by MS of a single protein with multiple peptides or the spot has been identified by Western blot. ii) '*': identification of the spot inferred from MS and/or 2D-PAGE information indicate only one potential protein. iii) '/': identification of the spot inferred from MS and/or 2D-PAGE information indicate more than one potential protein. iv) the protein in the spot cannot be identified or predicted by our techniques.
(XLSX)

S4 Table Spots of interest Mascot search information. This table provides Mascot search information of identified proteins. Four levels of identification are used and each one coded by a label: i) '$': identification by MS of a single protein with multiple peptides or the spot has been identified by Western blot. ii) '*': identification of the spot inferred from MS and/or 2D-PAGE information indicate only one potential protein. iii) '/': identification of the spot inferred from MS and/or 2D-PAGE information indicate more than one potential protein. iv) the protein in the spot cannot be identified or predicted by our techniques.
(XLSX)
