# Supplementary figures and images for: Proteomic analysis of human epileptic neocortex predicts vascular and glial changes in epileptic regions
Source: PLoS One. 2018 Apr 10;13(4):e0195639. doi: 10.1371/journal.pone.0195639 (PMC5892923; doi:10.1371/journal.pone.0195639)

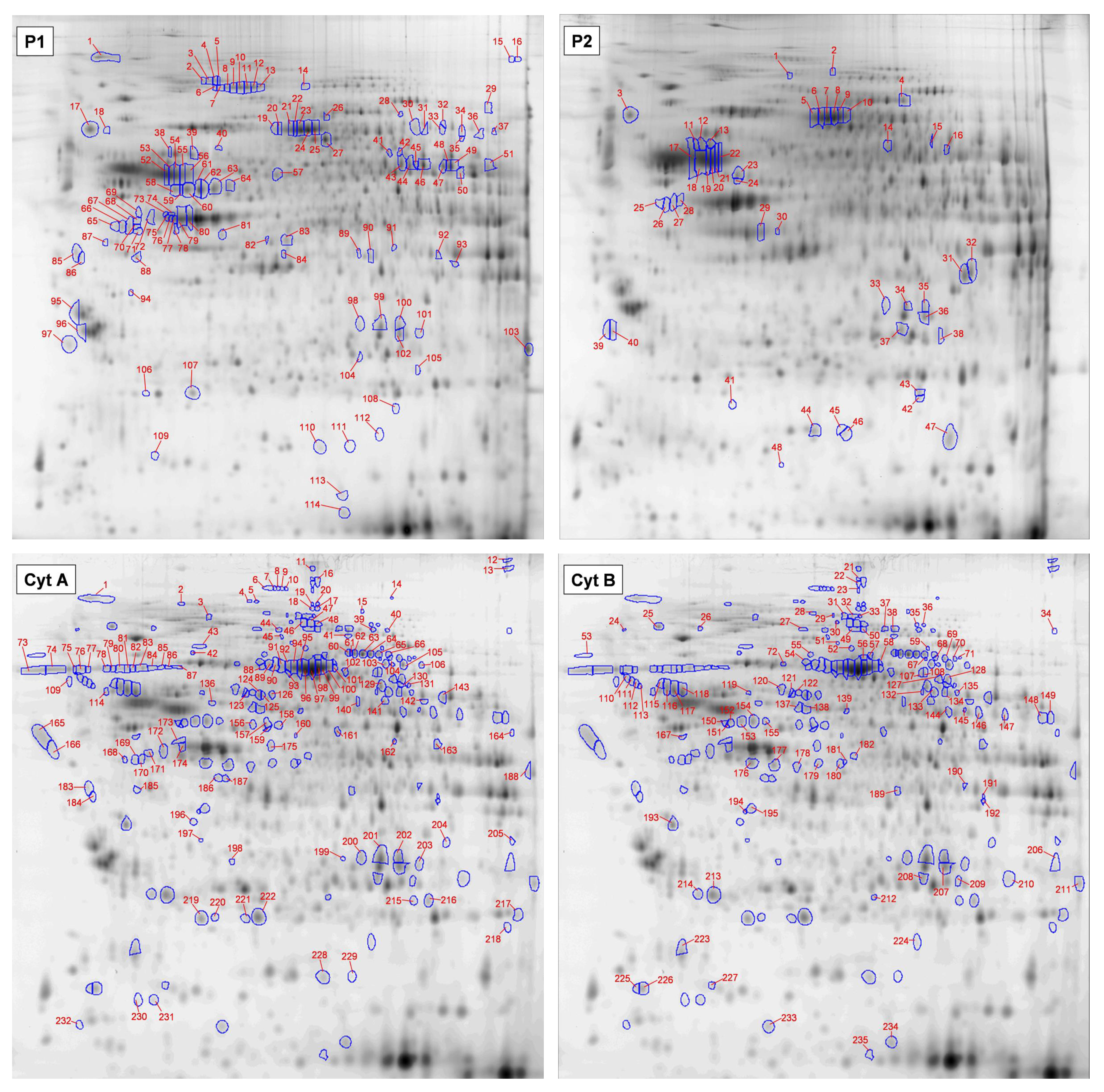

Supplement: S1 Fig — Each image represents 2D-PAGE of proteins from subcellular fractions using pH range of 3–11 (non-linear) and MW range 200-15kDa. SOIs are marked by blue perimeter and spot number. Note—the SOIs numbers in the cytosolic fraction are split across two images (CytA & CytB) in order to allow their visualization. P1 is the nuclear fraction and P2 is the membrane fraction. CytA and CytB are identical images of the cytosolic fraction used to accommodate different SOI numbers. (TIF) [file pone.0195639.s001.tif]

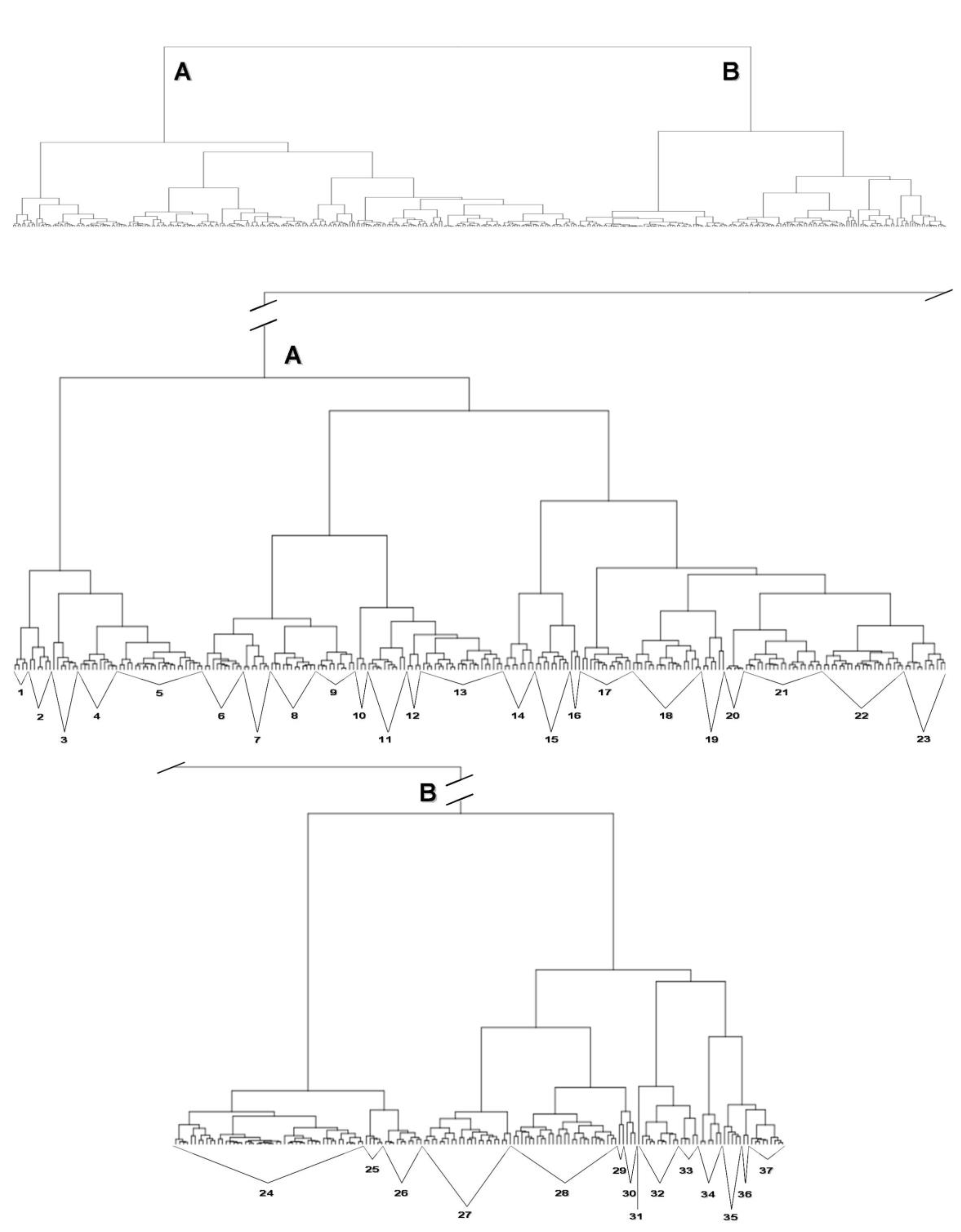

Supplement: S2 Fig — Visualization of hierarchical clustering of 397 SOIs by their expression patterns. The dendrogram was divided into two main branches (A & B) and an enlarged image of each branch is shown. An arbitrary distance cutoff was used to separate the dataset to 37 groups of spots. (TIF) [file pone.0195639.s002.tif]
